# Whole-genome resequencing of Hanwoo (Korean cattle) and insight into regions of homozygosity

**DOI:** 10.1186/1471-2164-14-519

**Published:** 2013-07-30

**Authors:** Kyung-Tai Lee, Won-Hyong Chung, Sung-Yeoun Lee, Jung-Woo Choi, Jiwoong Kim, Dajeong Lim, Seunghwan Lee, Gul-Won Jang, Bumsoo Kim, Yun Ho Choy, Xiaoping Liao, Paul Stothard, Stephen S Moore, Sang-Heon Lee, Sungmin Ahn, Namshin Kim, Tae-Hun Kim

**Affiliations:** 1 Animal Genomics and Bioinformatics Division, National Institute of Animal Science, Rural Development Administration, Suwon 441-706, Republic of Korea; 2 Korean Bioinformation Center, Korea Research Institute of Bioscience and Biotechnology, Daejeon 305-806, Korea; 3 Lee Gil Ya Cancer and Diabetes Institute, Gachon University of Medicine and Science, Incheon, Korea; 4 Department of Agricultural, Food and Nutritional Science, University of Alberta, Edmonton, AB T6G 2P5, Canada; 5 Hanwoo Experiment Station, National Institute of Animal Science, Rural Development Administration, Gangwon-do 323-950, Korea; 6 Animal Breeding and Genetics Division, National Institute of Animal Science, Rural Development Administration, Cheonan 331-801, Korea; 7 Queensland Alliance for Agriculture & Food Innovation, University of Queensland, St Lucia, Queensland, Australia; 8 Department of Bioinformatics, University of Science and Technology, Daejeon 305-806, Korea

**Keywords:** Hanwoo, Resequencing, NS/SS/I, ROH

## Abstract

**Background:**

Hanwoo (Korean cattle), which originated from natural crossbreeding between taurine and zebu cattle, migrated to the Korean peninsula through North China. Hanwoo were raised as draft animals until the 1970s without the introduction of foreign germplasm. Since 1979, Hanwoo has been bred as beef cattle. Genetic variation was analyzed by whole-genome deep resequencing of a Hanwoo bull. The Hanwoo genome was compared to that of two other breeds, Black Angus and Holstein, and genes within regions of homozygosity were investigated to elucidate the genetic and genomic characteristics of Hanwoo.

**Results:**

The Hanwoo bull genome was sequenced to 45.6-fold coverage using the ABI SOLiD system. In total, 4.7 million single-nucleotide polymorphisms and 0.4 million small indels were identified by comparison with the Btau4.0 reference assembly. Of the total number of SNPs and indels, 58% and 87%, respectively, were novel. The overall genotype concordance between the SNPs and BovineSNP50 BeadChip data was 96.4%. Of 1.6 million genetic differences in Hanwoo, approximately 25,000 non-synonymous SNPs, splice-site variants, and coding indels (NS/SS/Is) were detected in 8,360 genes. Among 1,045 genes containing reliable specific NS/SS/Is in Hanwoo, 109 genes contained more than one novel damaging NS/SS/I. Of the genes containing NS/SS/Is, 610 genes were assigned as trait-associated genes. Moreover, 16, 78, and 51 regions of homozygosity (ROHs) were detected in Hanwoo, Black Angus, and Holstein, respectively. ‘Regulation of actin filament length’ was revealed as a significant gene ontology term and 25 trait-associated genes for meat quality and disease resistance were found in 753 genes that resided in the ROHs of Hanwoo. In Hanwoo, 43 genes were located in common ROHs between whole-genome resequencing and SNP chips in BTA2, 10, and 13 coincided with quantitative trait loci for meat fat traits. In addition, the common ROHs in BTA2 and 16 were in agreement between Hanwoo and Black Angus.

**Conclusions:**

We identified 4.7 million SNPs and 0.4 million small indels by whole-genome resequencing of a Hanwoo bull. Approximately 25,000 non-synonymous SNPs, splice-site variants, and coding indels (NS/SS/Is) were detected in 8,360 genes. Additionally, we found 25 trait-associated genes for meat quality and disease resistance among 753 genes that resided in the ROHs of Hanwoo. These findings will provide useful genomic information for identifying genes or casual mutations associated with economically important traits in cattle.

## Background

The bovine genome was one of the first mammalian genomes sequenced, likely because cattle are important farm animals serving as major nutritional sources for humans and because of their evolutionary position as a representative of the Ruminantia, a phylogenetically distant clade to humans and rodents [1]. The bovine genome sequencing consortium sequenced a single inbred female Hereford cow and her sire using a combination of hierarchical sequencing and whole-genome shotgun sequencing [2]; the data have been assembled into two reference genomes, Btau and UMD [3,4].

After the bovine reference genome was assembled, several bovine genomes were resequenced, providing more insight into the genetic diversity of cattle that may be associated with phenotypic differences between breeds. In 2008, Van Tassell *et al*. reported more than 60,000 putative single-nucleotide polymorphisms (SNPs) identified from a reduced representation DNA library of 66 cattle representing three populations [5]. In 2009, Eck *et al*. performed the first single cattle whole-genome resequencing and reported more than 2 million novel SNPs in a Fleckvieh bull [6]. In 2011, Kawahara-Miki *et al*. resequenced the genome of a single Kuchinoshima-Ushi bull, a Japanese native cattle breed whose lineage has been strictly maintained in a small island secluded from mainland Japan [7]. In that study, more than 5.5 million novel SNPs were reported, and the Kuchinoshima-Ushi bull was determined to be genetically distinct from European domestic cattle breeds. Most recently, Stothard and colleagues reported whole-genome resequencing of Black Angus and Holstein, representative beef and dairy breeds, respectively, in North America, leading to the identification of substantial numbers of SNPs and copy number variants (CNVs) that could potentially be used as genetic markers across the genome [8].

When high-density genome-wide SNP data are available, analyses can identify genetic differences between similar populations. Understanding the genetic mechanisms leading to phenotypic differentiation requires identification of the genomic regions that have been under artificial selection in cattle breeds. For example, strong artificial selection will increase the frequency of favorable alleles at loci affecting meat quality traits in meat-producing breeds such as Hanwoo or Black Angus. In this process, a small region of the genome surrounding the mutations is also selected, resulting in a small genome region that shows reduced variation. Many methods have been developed for the detection of selection signatures from genome analyses, such as the use of regions of homozygosity (ROHs) [9], the integrated haplotype score (iHS) [10], *F*_ST_[11], and the extended haplotype homozygosity (EHH) statistic [12], according to the detection of the timescale for selection signatures. ROHs are without heterozygosity in the diploid states and provide association evidence at the genome-wide scale for complex traits.

Hanwoo, a Korean cattle breed, is reported to have originated from crossbreeding between taurine and zebu cattle and migrated to the Korean peninsula through North China; their history as a draft animal dates back at least 5,000 years [13,14]. Afterward, Hanwoo was maintained without the introduction of additional germplasm. Hanwoo was raised as a draft animal until the 1970s. In the late 1970s, the Korean government initiated a Hanwoo genetic breeding program to improve meat quantity and quality.

In this study, we sequenced the genome of a Hanwoo breeding bull and identified single nucleotide polymorphisms (SNPs) based on the *Bos taurus* reference genome assembly (Btau4.0). SNPs of Hanwoo were compared with those of Black Angus and Holstein. Moreover, functional annotation was carried out for SNPs. We also investigated genomic regions of homozygosity in Hanwoo, Black Angus, and Holstein.

## Results and discussion

### Genome sequencing, SNP/indel detection, and genotype concordance

Whole-genome sequencing of a Hanwoo bull was performed using the ABI SOLiD platform. Approximately 6.04 billion reads were produced from three independently prepared libraries. Using BFAST, ~3.68 billion reads of 120 gigabases (Gb) were aligned to Btau4.0 and filtered for redundant sequence reads. In total, 98.3% of the reference genome sequence was covered with an average mapping depth of ~45.6-fold (Table 1, Additional files 1, 2 and 3). This up-to-date Hanwoo sequence coverage was the highest in the bovine genomes sequenced until now, which could facilitate more reliable SNP identification [6-8]. Sequencing data from Black Angus and Holstein were reanalyzed with modified parameters to compare the sequencing data of Hanwoo to Black Angus and Holstein from a previous report [8]. The mapping depth of coverage in the Black Angus and Holstein were 9.8-fold and 10.8-fold, respectively, slightly lower than that in a previous report [8]. This inconsistency may be due to a difference in the application programs and algorithms used for analysis. However, in spite of relatively low read depths of Black Angus and Holstein bulls, 97.4% and 97.7% of the reference genome was covered by the sequenced reads at the minimum read depth of 1, respectively (Additional file 3), higher than the 93% coverage with 15.8-fold mapping depth reported in Kuchinoshima-Ushi [7].

**Table 1:** Summary of the sequenced reads for Hanwoo, Black Angus, and Holstein

	**Library**		**Number of total reads**	**Number of mapped reads**	**Total bases of mapped reads**	**Mean depth**	**Genome coverage (%)**
Hanwoo	MP^1^	2×50	6041252247	3680657166	120 Gb	45.6 **X**	98.3
	MP	2×50					
Black Angus	MP	2×25	2981165000	928443599	25.9 Gb	9.8 **X**	97.4
	FL^2^	1×50					
	MP	2×50					
Holstein	MP	2×25	2041487844	764185637	28.4 Gb	10.8 **X**	97.7
	FL	1×50					

^1^*MP* mate-paired library.

^2^*FL* fragment library.

In total, 4,781,758 SNPs were identified in the Hanwoo genome using the Genome Analysis Tool Kit (GATK) 1.0.5974 [15,16]. Among them, 2,327,616 SNPs (48.8%) were found in the single-nucleotide polymorphism database (dbSNP, build 133) while the remaining 2,454,142 SNPs (51.2%) were novel; 3,104,888 (64.9%) were heterozygous and 1,676,870 (35.1%) were homozygous, with a ratio of 1:1.85 (homozygous:heterozygous) (Additional file 3). Using UnifiedGenotyper in GATK, we identified 391,512 small indels (−14 to +22bp); 228,121 (58.3%) were heterozygous and 163,391 (41.7%) were homozygous (160,316 insertions and 231,196 deletions). Of these indels, 49,225 were found in dbSNP (build 133) while the remaining 342,287 indels (87.4%) were novel. All SNPs and indels identified in Hanwoo were submitted to the dbSNP at NCBI under the handle NIAS_AGBSGL.

To evaluate the SNP calling from our high-throughput genome sequencing data, concordance analysis was performed between Hanwoo genome resequencing and the SNP chip data. The same genomic DNA from Hanwoo used for deep resequencing was genotyped for 54,001 SNPs using BovineSNP50 BeadChip (Illumina). All probe sequences were mapped against the Btau4.0 reference genome assembly, and 50,411 positions were identified as unique genomic loci. In total, 1,061 (2.8%) of 38,049 homozygous calls by the SNP chip have been identified as heterozygous by NGS. In total, 526 (4.3%) of 12,362 heterozygous calls by the SNP chip were identified as homozygous by NGS (Additional file 4). The overall genotype concordance was 96.2%. The non-reference sensitivity and non-reference discrepancy rates were 97.1% and 7.0%, respectively. Non-reference sensitivity is the fraction of sites called variants (A/B or B/B) in comparison to those that are also called variants in evaluation data. The non-reference discrepancy rate, which is a good measure for testing the accuracy of genotype calls, can show the accuracy of genotype calling at sites called by both sites by excluding concordant genotypes (http://gatkforums.broadinstitute.org/discussion/48/using-varianteval).

### Functional annotation of genomic variation

**Figure 1:**
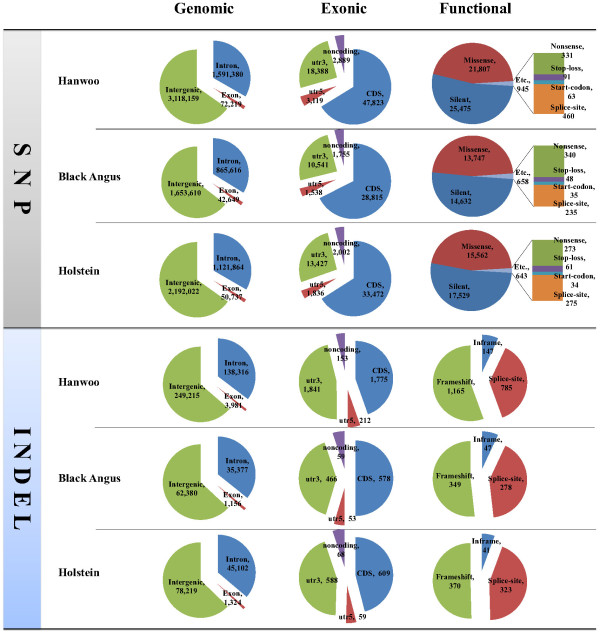
Genetic variations in Hanwoo, Black Angus, and Holstein.

The SNPs in genic regions were annotated using 20,955 genes from the NCBI Reference Sequence Database (RefSeq). In total, 1,663,599 SNPs (34.8%) identified in the Hanwoo genome were located in genic regions: 1,591,380 SNPs were located in introns, 21,507 SNPs were located in untranslated regions (UTRs), and 460 SNPs were located in splice sites. In total, 47,823 coding SNPs including 22,752 non-synonymous nucleotide substitutions such as missense and nonsense/read-through SNPs were also found (Figure 1 and Additional file 3). In total, 142,297 indels (36.4%) were in genic regions, of which 2,163 indels were identified as variations that may change amino acid sequences such as frameshift, nonsense, and splice-site SNPs, which may have the potential to cause functional differences. Non-synonymous SNPs, splice-site variants, and coding indels within a coding DNA sequence (NS/SS/I), which may affect gene function, were detected in Hanwoo (24,915 in 8,360 genes), Black Angus (15,107 in 6,563 genes), and Holstein (16,963 in 6,692), respectively (Additional files 3 and 5). The Hanwoo genome contained more NS/SS/Is than those of Black Angus and Holstein. This suggests that Hanwoo is a more genetically distant breed than Black Angus and Holstein based on the reference genome of Hereford, which is consistent with a previous report [17]. Of all reference genes (20,955), 10,906 genes contained NS/SS/I genes and 737 genes revealed more than 10 NS/SS/Is in all breeds (Additional file 5). ATP-binding cassette subfamily C member 4 (*ABCC4*) and zinc-finger protein 280B (*ZNF280B*) genes showed more than 100 NS/SS/Is. Four isoforms (copies) of the *ABCC4* gene are located on BTA12 in tandem with each other (ENSBTAG00000032603, ENSBTAG00000047764, ENSBTAG00000023309, and ENSBTAG00000047383). Fifty-four variations (NS/SS/Is) in four isoforms are recorded in Ensembl. However, the *ZNF280B* gene is a single-copy gene (ENSBTAG00000001005) located on BTA17 and 83 NS/SS/Is exist in Ensembl, although *ZNF280B* has a smaller genome span (8.463 kb) and transcript (1.980 kb) compared to the genome spans (87.521 to 165.199 kb) and transcripts (2.529 to 3.930 kb) of *ABCC4* gene copies. These findings show that these two genes surely belong to the gene group of more NS/SS/Is rather than other common genes. A study has reported that the number of copies of the *ABCC4* gene increases and the gene is overexpressed in the process of selection for resistant mouse cells against antibiotics such as ciprofloxacin [18]. Therefore, this suggests that genes containing several NS/SS/Is may have evolved into multi-copy genes for environmental adaptation, or that NS/SS/Is may be distorted due to an incorrect reference genome sequence. However, this is necessary for experimental validation based on phenomena such as CNV or segmental duplication. Alternatively, the possibility of the presence of pseudogenes should not be excluded for genes containing several NS/SS/Is. Among 10,906 genes containing NS/SS/Is, the number of genes containing specific NS/SS/Is was 1,983 in Hanwoo, 1,199 in Black Angus, and 900 in Holstein. In Hanwoo, 1,045 genes contained reliable specific NS/SS/Is with more than tenfold depth. Furthermore, of 1,045 genes containing specific NS/SS/Is, 293 genes were revealed in Hanwoo only and 109 genes contained more than one novel damaging NS/SS/I in the functions among them (Additional file 6). Seven NS/SS/Is and six novel damaging NS/SS/Is were found in Hanwoo specifically within the raftlin lipid raft linking protein 1 (*RFTN*1) gene, which is important in the formation or maintenance of membrane lipid rafts [19] and is overexpressed in smooth muscles (Gene Expression Atlas in EBI).

Next, we investigated whether NS/SS/I-containing genes were associated with economic traits and then categorized them into meat, disease resistance, growth, milk, and fecundity. We used previously reported information on trait-associated genes [7,20,21]. In total, 619 genes were assigned as trait-associated genes: 464 genes for meat quality, 144 genes for disease resistance, 25 genes for milk production, 8 genes for fecundity, and 6 genes for growth rate (Additional file 5). Of the 464 genes for meat quality, 228 contained more than one NS/SS/I. The titin (*TTN*) gene has 62 NS/SS/Is, the largest number among the genes related to meat quality. The bovine major histocompatibility complex (MHC) class I heavy chain isoform 1 precursor (*BOLA*, ENSBTAG00000002069) gene, which contain 32 NS/SS/Is, has the largest number of NS/SS/Is among the genes related to disease resistance (Additional file 5). The higher number of NS/SS/Is in *TTN* than *BOLA* may be due to the difference of gene size; 274.866 kb and 3.788 kb of TTN and BOLA genes, respectively. The *TTN* gene encodes the titin protein, the largest protein, which consists of 317 exons in 274.866 kb of genomic DNA in BTA2 (Ensembl database UMD3.1). The *TTN* gene plays a role in myofibrillogenesis and is associated with marbling [22]. Moreover, the 231054C>T variant within the promoter region of *TTN* is associated with a marbling trait and is differentially expressed between high- and low-marbling muscle samples [23]. However, many NS/SS/Is likely affect the function of titin, which acts as a molecular spring for the passive elasticity of muscles [24]. These NS/SS/Is within the *TTN* gene may be informative variants for understanding the effects of steric changes in the TTN protein. Of the 144 genes for disease resistance, 74 also contained NS/SS/Is, and many novel damaging NS/SS/Is were detected in several genes including BCL2-like 1 (*BCL2L1*), nitric oxide synthase 1 (*NOS1*), nucleotide-binding oligomerization domain-containing protein 2 (*NOD2*), granzyme A (*GZMA*), and semaphorin-5A (*SEMA5A*), as well as the *BOLA* gene (Additional file 5). Among 109 genes containing more than one novel damaging specific NS/SS/Is in Hanwoo, the *BCL2L1*, *GZMA*, and *CD5* genes are known as candidate genes for the disease resistance trait (Additional file 6). We suggest that the exonic variation identified in this study will provide valuable information for functional studies as well as marker development associated with economic traits in cattle.

### Regions of homozygosity within the three breeds

A ROH is a continuous or uninterrupted stretch of DNA without heterozygosity in the diploid state. A discrepancy has existed in the minimum standard of definition of ROH among the groups that have been studied for ROHs to date [25]. Most previous ROH studies have been performed with SNP chip results, an average of 50 SNPs of 5 Mb in size with an average distance of 100 kb between them, and an allowance of up to 2% heterozygous SNPs within a ROH [25]. However, at present, no standardized criteria have been established for defining ROHs [25]. In this study, using mass genotype data derived from whole-genome resequencing, we shortened the detection window of ROHs and loosened the permissible ratio of heterozygous SNPs (Additional file 7).

**Figure 2:**
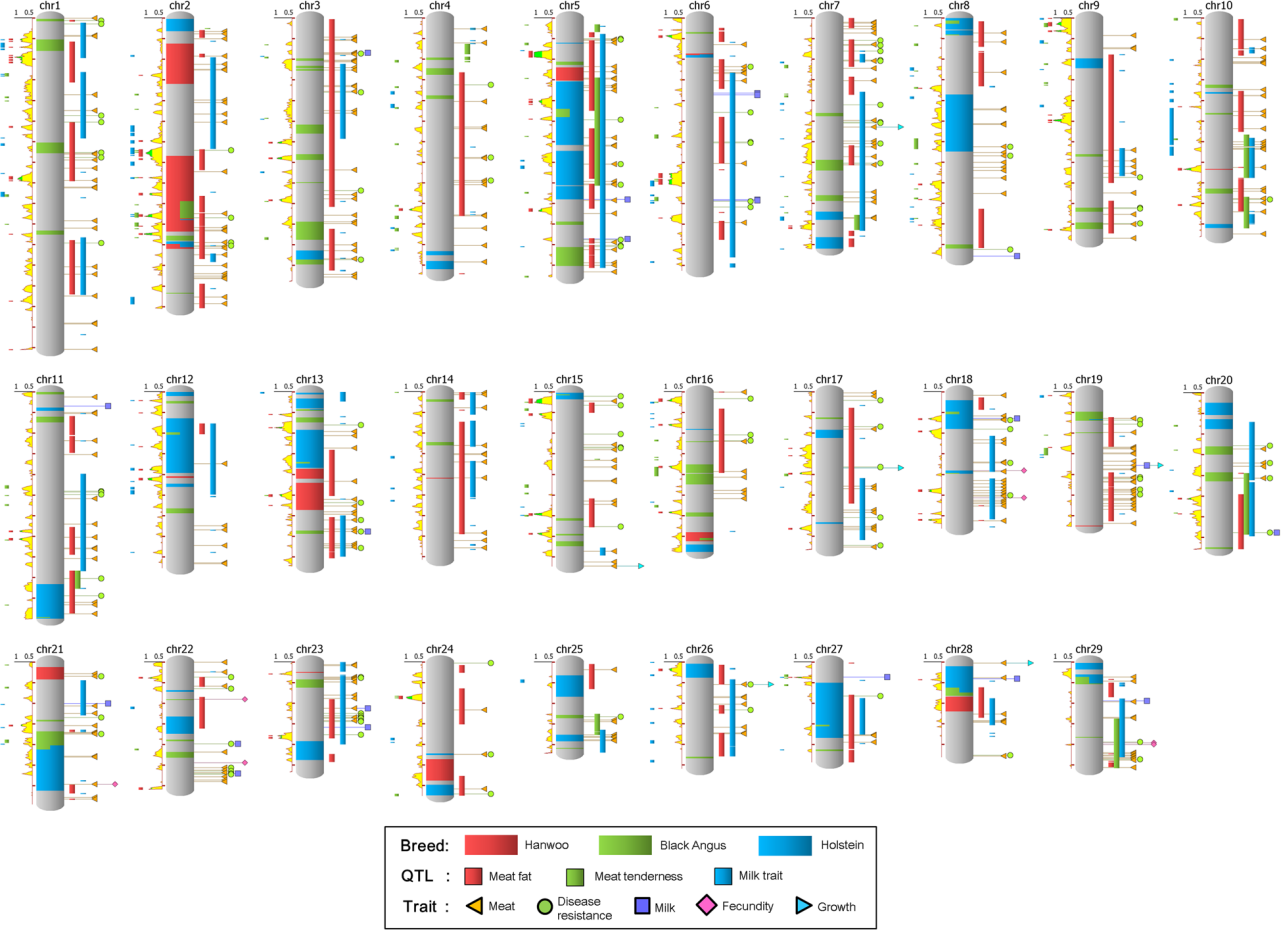
**Integrated ROH and QTL maps of *Bos taurus *chromosomes.** NGS ROHs (ROH defined by the SNP derived from NGS) are positioned onto a chromosome image. The left side of a chromosome shows Chip ROH (ROH defined by the SNP derived from the SNP chip) information. The line chart at the left of a chromosome indicates the Hanwoo homozygosity ratio defined by the SNP chip; ratios from 0.5 to 0.8 are shaded in yellow and ratios >0.8 are shaded in green. The bars at the left of the line chart indicate the Chip ROH. The right side of a chromosome shows QTL and trait-associated genes. The bars at the right of a chromosome indicate the genomic positions of meat and milk-related QTLs. At the right of the QTL bars, five types of trait-associated genes point to their genomic locations.

Our criteria were as follows: the ROH detection window was 400 kb and 20% of heterozygous SNPs were allowed for Hanwoo, Black Angus, and Holstein (Figure 2). We defined 16, 78, and 51 ROHs in Hanwoo, Black Angus, and Holstein, respectively (Table 2 and Additional file 8). Angus and Holstein were bred for meat and milk production, respectively. In contrast, Hanwoo was raised as a draft animal until the 1970s. Since 1979, Hanwoo has been bred as beef cattle according to the Hanwoo genetic improvement national program organized by the government. Here, we suggest that the total lengths of ROHs in Holstein and Black Angus are longer than those of Hanwoo because Holstein and Black Angus have been artificially selected for a longer period of time. Overall, the dispersing pattern of ROHs in chromosomes was variable and also differed in the overlapping pattern of ROHs between breeds; we found two overlapping regions between Hanwoo and Black Angus, three overlapping regions between Hanwoo and Holstein, and 14 overlapping regions between Black Angus and Holstein (Additional file 8). These patterns would result from different origins and breeding strategies among the three breeds because Black Angus and Holstein originated in Aberdeen, Scotland and the Netherlands, respectively, and have been bred as beef and dairy cattle, respectively, while Hanwoo was bred independently as beef cattle in the Korean peninsula since 1979.

**Table 2:** Summary of specific regions of homozygosity (ROHs) in Hanwoo, Black Angus, and Holstein

	**Breed**	**No. of ROHs**	**ROH size (Mb)**	**Average ROH size (Mb)**	**Gene count**	**Trait-associatedgene count**	**NS/SS/I**^**2 **^**(novel)**
**ROH**	Hanwoo	16	114.4	7.15	753	24	1,133 (762)
	Black Angus	78	176.8	2.26	1321	46	1,946 (1,289)
	Holstein	51	354	6.94	2483	68	3,905 (2,657)
**sROH**^**1**^	Hanwoo	19	102.4	5.39	650	19	968 (675)
	Black Angus	67	147.6	2.2	1109	41	1,625 (1,087)
	Holstein	56	332	5.93	2322	64	3,693 (2,512)

^1^ sROH: ROH specific to a breed identified by comparing ROHs among breeds. A single ROH can produce more than one sROH when a middle portion of the ROH is present in another breed such that the two outer regions of the ROH are specific to a breed. Alternatively, if an entire ROH is present in more than one breed it is not counted as a sROH in any breed.

^2^*NS*/*SS*/*I* non-synonymous SNP/splice-site variation/coding indel.

A total of 753 genes resided in the ROHs of Hanwoo, whereas 1,320 and 2,482 genes existed in the ROHs of Black Angus and Holstein, respectively. Among them, 77 and 30 common genes were located within the overlapping ROHs between Hanwoo and Angus and between Hanwoo and Holstein, respectively (Additional file 9). Among the 753 genes in the ROHs of Hanwoo, 505 genes contained no NS/SS/Is. A total of 2,158 genes (10.2%) contained no NS/SS/Is in the ROHs in any of the three breeds (Additional file 5). Moreover, we performed functional enrichment analysis using gene ontology (GO) for genes in the ROHs of the three breeds (Additional file 10). In Hanwoo, one significant GO term was ‘regulation of actin filament length related to muscle metabolism’ (GO:0030832, p-value = 0.044), including actin-related protein 3 homolog (*ACTR3*), actin-related protein 2/3 complex, subunit 2 (*ARPC2*), villin 1 (*VIL1*), and destrin (*DSTN*) genes. Meat tenderness is generated by the disruption of actin filaments and by breaking down the interaction between the actin and myosin filaments [26]. Notably, a significant GO term of ‘striated muscle cell differentiation’ (GO:0051146, p-value = 0.034) was found in Holstein, including the retinoid X receptor, alpha (*RXRA*) gene, which inhibits adipogenesis [27] and plays a negative role in marbling in Hanwoo [28]. Because Hanwoo and Black Angus were bred as beef cattle, 77 genes in overlapping ROHs between the breeds were used to analyze GO and the KEGG pathway. Although eight significant GO terms were detected, most were related to the immune system, such as T cell activation and lymphocyte activation, rather than meat traits. The presence of many immune system-related genes in the identified ROHs could reflect selection (natural or artificial) for disease resistance. According to functional enrichment analysis using KEGG pathway terms, vitamin B6 metabolism (bta00750, p-value = 0.025) was significantly enriched, including the aldehyde oxidase 1 (*AOX1*) gene in Hanwoo (Additional file 10). Vitamin B6 induces the differentiation of adipocytes from pre-adipocytes and facilitates fat accumulation [29]. In particular, the *AOX1* gene is a target of peroxisome proliferator-activated receptors alpha and gamma (*PPARα* and *PPAR*γ) as a key gene in adipogenesis [30]. Melanogenesis (bta04916, p-value = 0.009) was also detected in Holstein. Sixteen genes, including tyrosinase (*TYR*) and the melanocortin 1 receptor (*MC1R*), exist in this term. TYR is the rate-limiting enzyme in the melanogenesis pathway. Tyrosinase activity is regulated by the MC1R. Recently, Kuhn and Weikard reported the dilution of black pigment (eumelanin) in an F_2_ Holstein × Charolais population [31]. These genes may also be partially responsible for the coat color pattern of Holsteins. These observations suggest that genes within the ROH accumulate the biological functions for characteristics of each breed during a process of artificial selection.

We found trait-associated genes containing NS/SS/Is in the ROHs of each breed (Table 3). Twenty-five trait-associated genes for meat quality and disease resistance were found in the ROHs of Hanwoo. Among these, nine were associated with meat quality traits and contained NS/SS/Is: *TTN*, acetylcholine receptor subunit alpha (*CHRNA1*), isocitrate dehydrogenase 1 (*IDH*), amylotrophic lateral sclerosis 2 (*ALS2*), Sp1 transcription factor (*SP1*), retinoic acid receptor gamma (*RARG*), collagen type IX alpha 3 (*COL9A3*), fatty acid binding protein 4 (*FABP4*), and insulin-like growth factor 1 receptor (*IGF1R*; Table 3 and Additional file 5). In humans, the homozygosity association approach has been applied to identify causal mutations for autosomal recessive disorders in consanguineous families [32-40], as well as the genome-wide investigation of candidate genes for complex phenotypes such as schizophrenia [41], late-onset Alzheimer’s disease [42], and height [43]. Unlike humans, farm animals are artificially selected to improve genetic performance for economic traits such as meat quality and milk production. Therefore, genes related to economically important traits are gradually fixed with a dominant allele as a result of artificial selection. In livestock, fixed genes should be considered major genes that control a certain trait for any breed because genes can become fixed, dominant alleles through artificial selection or evolution. However, we suggest that genes containing NS/SS/Is in ROHs are still strong candidate genes for meat quality traits in the Hanwoo population because they may be in the selection process for breeding. According to previous reports, a splice-site mutation within the *FABP4* gene in Australian cattle is significantly associated with intramuscular fat content [44]; SNPs within the *FABP4* gene are associated with palmitoleic acid and linoleic acid content in intramuscular fat in Japanese cattle [45] and with backfat thickness [46], marbling score, and carcass weight in Hanwoo [47]. The *IDH1* gene, which is responsible for ketoglutarate, CO_2_, and NADPH production from isocitrate in the cytosol and associated with body weight and fat deposition [48], had one damaging NS/SS/I in Hanwoo.

**Table 3:** Trait-associated genes in ROHs of Hanwoo, Black Angus, and Holstein

**Breed**	**ROH_ID**	**Chr.**	**Gene no.**	**Trait_ID**	**Trait-associated genes**
Hanwoo	HW_ROH_1	2	118	MT^1^	*PDK1*, *HAT1*, *TTN*, *CHRNA1*
	HW_ROH_2	2	152	MT, DR^2^	*DBI*, *CTLA4*, *CD28*, *IDH1*, *ALS2*, *MYL1*, *ACADL*
	HW_ROH_3	2	63	MT, DR	*PRKAG3*, *DES*, *SLC11A1*
	HW_ROH_4	5	98	MT	*SP1*, *RARG*, *SOCS2*
	HW_ROH_9	13	150	MT, DR	*COL9A3*, *SIRPA*, *ATRN*
	HW_ROH_10	14	5	MT	*FABP4*, *FABP5*
	HW_ROH_13	21	21	MT, DR	*MEF2A*, *IGF1R*
Black Angus	BA_ROH_1	1	19	DR	*IFNAR1*, *IL10RB*
	BA_ROH_3	1	21	DR	*CD80*
	BA_ROH_5	2	64	MT, DR	*CTLA4*, *CD28*, *ALS2*
	BA_ROH_8	3	26	MT	*RORC*
	BA_ROH_9	3	26	MT	*PRKAB2*
	BA_ROH_10	3	8	MT	*NOTCH2*
	BA_ROH_14	3	93	MT	*CPT2*, *DIO1*
	BA_ROH_15	3	26	DR	*PSMB2*
	BA_ROH_22	5	169	MT	*SREBF2*, *FGF6*, *MCHR1*
	BA_ROH_25	7	30	MT, DR	*ADRA1B*, *IL12B*
	BA_ROH_26	7	6	MT	*COX7C*
	BA_ROH_31	9	12	DR	*ESR1*
	BA_ROH_32	9	25	MT, DR	*IGF2R*, *SOD2*
	BA_ROH_35	10	20	MT	*ACTN1*, *SLC8A3*
	BA_ROH_45	13	18	MT, DR, MK^3^	*LBP*, *TGM2*
	BA_ROH_52	16	5	MT	*CAPN2*
	BA_ROH_53	16	40	MT	*PLOD1*
	BA_ROH_54	16	58	MT	*SLC2A5*
	BA_ROH_60	19	55	MT, DR	*CCL3*, *ACACA*, *CCL5*
	BA_ROH_61	19	20	MT	*VTN*
	BA_ROH_62	20	14	MT	*FST*
	BA_ROH_63	20	33	MT, DR	*PRLR*
	BA_ROH_66	21	48	MT, DR	*CYP1A1*, *CTSG*, *GZMH*, *GZMB*, *LOXL1*
	BA_ROH_67	22	5	FE^4^	*OXTR*
	BA_ROH_69	22	28	MT	*PDHB*
	BA_ROH_70	23	56	MT	*PPARD*, *COX5B*
	BA_ROH_71	25	9	DR	*IL4R*
	BA_ROH_77	29	20	MT	*CTSC*, *ME3*
Holstein	HO_ROH_3	2	35	MT, DR	*IGFBP2*, *PRKAG3*, *SLC11A1*, *IGFBP5*
	HO_ROH_4	3	60	MT	*COL8A2*, *COL9A2*
	HO_ROH_6	4	27	MT	*PRKAG2*
	HO_ROH_7	5	5	MT	*MYF5*, *MYF6*
	HO_ROH_8	5	292	MT, DR	*PRKAG1*, *WNT1*, *SLC16A7*, *IFNG*, *PFKM*, *GPD1*
	HO_ROH_9	5	137	MT, DR	*CSF2RB*, *IGF1*, *MB*, *PMCH*, *PVALB*, *CACNG2*
	HO_ROH_12	7	23	MT	*CAST*, *PCSK1*
	HO_ROH_15	8	22	MT	*CTSB*, *FDFT1*
	HO_ROH_16	8	160	MT, DR	*ANXA1*, *VLDLR*, *CD72*, *TPM2*, *SMARCA2*, *ALDH1A1*
	HO_ROH_21	11	289	MT, DR	*PSMB7*, *CRAT*, *NOTCH1*, *ENG*
	HO_ROH_23	12	199	MT	*SGCG*
	HO_ROH_28	13	100	MT	*ITGB1*, *NEBL*, *PHYH*
	HO_ROH_29	15	11	DR	*CASP4*, *CASP1*
	HO_ROH_34	18	132	MT, DR, MK	*MC1R*, *CYBA*, *CDH15*, *FOXC2*
	HO_ROH_35	18	26	MT, FE	*HP*, *CALB2*
	HO_ROH_39	21	156	MT, FE	*SERPINA1*, *PSMA6*, *CFL2*, *SERPINA14*
	HO_ROH_43	24	13	MT, DR	*MC2R*, *MC5R*
	HO_ROH_44	24	54	MT, DR	*MC4R*, *BCL2*
	HO_ROH_46	25	87	MT	*ELN*, *HSPB1*, *PLOD3*, *GPC2*, *MDH2*, *SERPINE1*
	HO_ROH_48	27	134	MT, DR	*ADRB3*, *CASP3*, *FGFR1*
	HO_ROH_49	28	52	MT, MK	*MTR*, *ACTN2*
	HO_ROH_51	29	21	MT	*CTSC*, *ME3*

^1^*MT* meat traits.

^2^*DR* Disease-resistance traits.

^3^*MK* Milk traits.

^4^*FE* Fecundity traits.

In addition, to detect common ROHs between the genome sequence and SNP chip, we calculated the ROHs from 40 Hanwoo bulls as well as 20 Angus and 19 Holstein individuals using the Bovine 50K SNP chip (Figure 2). We identified four, eight, and eight common ROHs between both data sets in Hanwoo, Black Angus, and Holstein, respectively (Additional file 9). In Hanwoo, 43 genes located in common ROHs were shared between genome sequencing and SNP chips in BTA2, 10, and 13 (Figure 2). Of 43 genes, 22 genes contained no NS/SS/I (Additional file 5). Moreover, four common ROHs in Hanwoo coincided with quantitative trait loci (QTLs) for meat fat traits (Figure 2). Specifically, two regions in BTA2 (95.3-96.4 Mb and 100.9-101.4 Mb in Btau4.0) were common ROHs between genome sequencing and SNP chips in Hanwoo and Black Angus. Of the 18 genes that resided in these regions, WD repeat domain 12 (*WDR12*), amyotrophic lateral sclerosis 2 (juvenile) chromosome region, candidate 8 (*ALS2CR8*), cytochrome P450, family 20, subfamily A, polypeptide 1 (*CYP20A1*), and cAMP responsive element binding protein 1 (*CREB1*) genes belonged to a significant GO term of metabolic processes in Hanwoo. Among them, the *CREB1* gene has been shown to be related to fat metabolism. In 2012, Lee *et al*. reported that the expression of the cAMP responsive element binding protein (*CREB1*) gene is higher in muscle with high IMF content in Hanwoo [49]. CREB1 is a transcription factor containing a basic leucine zipper. The CREB protein is phosphorylated in response to increased cAMP, allowing it to efficiently interact with the transcriptional co-activator protein, CREB binding protein, to stimulate the transcription of cAMP target genes [50]. Moreover, Casimir and Ntambi reported that intracellular cAMP activates the expression of the *stearoyl*-*CoA desaturase* gene, a key enzyme involved in monounsaturated fatty acid synthesis through activation of the CREB protein [51]. In 2009, Wang *et al*. observed that messenger RNA expression of a lipogenesis-related gene, stearoyl-coA desaturase (SCD), peaked at 20 to 25 months in crosses between Wagyu and Hereford, which was highly correlated with intramuscular fat content in these animals [52]. These findings suggest that elevated CREB expression may stimulate genes involved in the lipid biosynthesis pathway such as SCD [51] and HMG-Co synthase [53], resulting in an increase in IMF content within muscles. Also, the *ALS2* gene, which is related to meat traits, as well as cytotoxic T-lymphocyte-associated protein 4 (*CTLA4*) and CD28 molecule (*CD28*) genes for disease resistance, resided in a common ROH (BTA2: 94.8-96.9 Mb in Btau4.0) in Hanwoo and Black Angus according to genome sequencing (Figure 2). In livestock animals bred by an improvement scheme for economic traits, the use of ROHs will be a good genomic strategy for tracking and planning improvements in breeding.

## Conclusions

In this study, we sequenced the whole genome of a Hanwoo bull and newly identified 2,454,142 SNPs and 342,287 small indels by comparison with the Hereford reference genome sequence. We also found 1,663,599 SNPs and 142,297 indels that were located in genic regions of 20,955 genes in the NCBI Reference Sequence Database (RefSeq), of which 22,752 SNPs and 2,163 indels were non-synonymous, frameshift, nonsense, or splice-site SNPs potentially capable of affecting protein functions. This suggests that genes containing several NS/SS/Is may have evolved into multi-copy genes for environmental adaptation, or that NS/SS/Is may be distorted due to an incorrect reference genome sequence. A ROH is a continuous or uninterrupted stretch of DNA without heterozygosity in the diploid state. In this study, we defined 16 ROHs in Hanwoo using a detection window of 400 kb and 20% of heterozygous SNPs using genotype data derived from whole-genome resequencing. The cumulative lengths of ROHs per genome, as well as the number of ROHs in Hanwoo, were smaller than those in Black Angus and Holstein. This suggests that the total lengths of ROHs in Holstein and Black Angus are longer than those of Hanwoo due to a longer period of time for artificial selection in those breeds. In addition, the dispersing pattern of ROHs in chromosomes was different between breeds. We suggest that these patterns would result from the different origins and breeding strategies among these three breeds. Moreover, 753 genes were observed in the ROHs of Hanwoo, of which 25 genes were associated with meat quality and disease resistance traits. In addition, we observed common ROHs between the genome sequence and high-density SNP chip data. This combinatorial ROH survey approach may be another effective method for identifying domestication genes. The findings of this study will provide valuable information for functional studies, as well as for marker development associated with economically important traits in cattle.

## Methods

### DNA samples

We sequenced the genome of a proven Hanwoo bull (27223) obtained from the Hanwoo Experiment Station, National Institute of Animal Science, Rural Development Administration, Korea. Bull 27223 was selected for mapping for its representativeness of the population at the Hanwoo Experiment station. Bull 27223 is a descendent of KPN369, which was one of the most frequently used Hanwoo bulls for artificial insemination in Korea during the early 2010s. Also, bull 27223 was selected for its superiority in growth performance with superior genetic potential in carcass quality. Therefore, many calves born since 2010 have been sired by this bull. The study protocol and standard operating procedures were reviewed and approved by the Institutional Animal Care and Use Committee of the National Institute of Animal Science (Suwon, Republic of Korea).

### Whole-genome sequencing library preparation

Genomic DNA (gDNA) was extracted from whole blood with a QIAamp DNA Blood Maxi Kit according to the manufacturer’s instructions (Qiagen). Libraries were prepared according to the SOLiD System Mate-paired Library Preparation protocol of the Applied Biosystems SOLiD System: Library Preparation Guide (02/2009 & 10/2009 editions).

Briefly, gDNA was fragmented using Covaris S2 (Covaris) and HydroShear (Genomic Solutions) at the proper settings for targeted sizes. A QIAquick Gel Extraction Kit (Qiagen) was used for subsequent purification of sheared DNA, enzymatic reactions, and size-selected DNA in agarose gels according to the manufacturer’s instructions. To repair damaged DNA ends and obtain 5′-phosphorylated blunt-ends (5′P), the fragments were end-repaired using the End-It DNA End-Repair Kit (Epicentre Biotechnologies) according to the manufacturer’s instructions. Ligations for the adaptor attachment and circularization were accomplished using the Quick Ligation Kit (New England BioLabs). DNA quantitations were performed using a NanoDrop ND 1000 Spectrophotometer (Thermo Fisher Scientific), except for those followed by library amplification for emulsion PCR (ePCR).

In chronological order, the sheared gDNA fragments were end-repaired and the LMP CAP Adaptors (missing the 5′ phosphate from one oligonucleotide resulting in a nick on each strand when the DNA is circularized at a later step) were ligated to the end-repaired DNA fragments. The adaptor-ligated products were separated on a 1% agarose gel and excised from the gel at the appropriate positions for span size ranges (600–700 bp, 1–2 kb, and 0.6-2.2 kb). Size-selected DNA fragments were circularized with a biotinylated internal adaptor. Uncircularized DNA fragments were eliminated using Plasmid-Safe ATP-Dependent DNase (Epicentre Biotechnologies). Nick translation was performed for 14 min at 0°C in an ice-water bath using *Escherichia coli* DNA polymerase I with the circularized DNA fragments. The nick-translated products were cleaved at the nicks using T7 exonuclease and S1 nuclease, and end-repaired as described above. P1 and P2 adaptors (used for library amplification, ePCR, and ligation sequencing) were ligated to ends of the end-repaired DNA. Then the ligated DNA underwent nick translation with DNA polymerase I. The completed library was amplified using Library PCR primers 1 and 2 with Cloned Pfu polymerase (Stratagene) or Platinum® PCR Amplification Mix (SOLiD Long Mate-Paired Library Construction Kit, ABI). The amplified library was ran on a 4% agarose gel and the correct-sized band (275–300 bp) was excised and eluted, and quantitated by Qubit IT (Invitrogen). ePCR was carried out according to the Applied Biosystems SOLiD System: Template Bead Preparation Guide. The concentration of each library for ePCR was designed to range from 1.0 to 1.5 pM.

### Library sequencing of template beads

Sequencing was performed according to the Applied Biosystems SOLiD System: Instrument Operation Guide. Templated beads were deposited onto two slides and sequencing was carried out to 50 bases using SOLiD v3.0 chemistry, with the exception that the library prepared from 0.6-2.2 kb-sheared DNA fragments was used for four slides and sequencing was carried out to 50 bases using SOLiD v3 plus chemistry.

### Short-read alignment, variant calling, and annotation

Paired-end 50 bp reads from Hanwoo, Black Angus, and Holstein were mapped to the Btau4.0 reference genome assembly using BFAST 0.7.0a [54], with options bfast match “-A 1 -z -K 100 -M 500,” bfast localalign “-A 1 -o 10,” and bfast postprocess “-A 1 -a 3 -Y 2 -z -O 1.” Aligned reads considered to be PCR duplicates were removed using the MarkDuplicates algorithm in Picard tools 1.57. This algorithm identifies the 5′ coordinates and mapping orientations of each read pair by considering gaps and jumps. The reads that mapped to the same position and orientation are marked as duplicates except the best scored read pair. The score of a read pair is defined as the sum of base qualities >15. Next, the IndelRealigner module in the Genome Analysis Toolkit (GATK) 1.0.5974 [15] was used to perform local realignment around indels to produce an accurate alignment and CountCovariates and TableRecalculation modules to recalibrate the base quality score. An in-house script was applied to modify the read quality, which was generated by BFAST before the GATK recalibration step. The quality scale generated by BFAST presented up to ~63 and was skewed to the maximum value. Such an overestimated quality scale prevented the filtration of false-positive variations while GATK runs genotyping. The in-house script scaled down the overestimated quality values to ~40. SNP and small indel calling were performed using GATK UnifiedGenotyper [16] with a minimum base quality of Q17 (phred score 17) with “--stand_call_conf 0 --stand_emit_conf 0 --max_deletion_fraction 1.00” and a minimum mapping quality of Q30 (phred score 30) with “--stand_call_conf 0 --stand_emit_conf 0 --genotype_likelihoods_model INDEL --minIndelCnt 3”. Hanwoo, Black Angus, and Holstein were genotyped separately using GATK UnifiedGenotyper. Then, the variants identified in three breeds were merged by genomic position for downstream analysis. A novel variant was defined as one that was not present in the cattle dbSNP 133.

Annotations of variants were based on the 34,577 Cow RefSeq in NCBI (downloaded April 2, 2012). The cattle RefSeqs were aligned against Btau4.0 using BLAT with the ‘fine’ option to obtain the genomic positions of genes, exons, and coding regions. In total, 33,080 RefSeqs were aligned against the reference genome. Among the aligned RefSeqs, the sequences with >90% coverage and a <1% error rate were selected. Then one representative RefSeq was selected from the RefSeqs derived from the same gene. As the result, we selected 29,197 RefSeqs for variant annotation. We identified 2-base canonical splice sites (GU/AG) at the end of an intron as a splice site. The genomic locations of some trait-associated genes that were not obtained from NCBI RefSeqs were defined from previously reported gene information [7]. The selected genes were used to predefine the annotation data of all possible variants and pre-calculate the SIFT [55] predictions and scores. We selected the coding indels, splice-site variants, and non-synonymous SNPs (NS/SS/Is) that showed SIFT scores of <0.05 as the potentially damaging variants.

Specific NS/SS/I variants were detected by the following criteria: We first selected the NS/SS/Is for which at least 10 reads were aligned and an allele was 50% more abundant than the other alleles for all three breeds at the position.

### ROHs

To measure the genome-wide pattern of selection of a breed, we defined a ROH as follows. The minimum ROH size was set to 400 kb; each chromosome was divided into 400 kb bins, and the ratio of homozygous SNPs per bin was employed as the degree of homozygosity of the bin. To look for a series of high-degree bins rather than separated, one-point peak bins, a degree was smoothed by an average of the two neighbor bins on each side. A continuous extension of bins with a high degree of homozygosity was defined as a ROH. In this study, a 0.8 degree was imposed to determine the ROHs. One breed may contain a ROH that shows a high degree of homozygosity while the others do not. This helps to explain the breed-specific selection pressure. We defined a subset of ROHs that was not duplicated in the other breed’s ROHs as specific ROHs (sROHs). ROHs were identified from SNP chip data using HomozygosityMapper [56].

### SNP genotyping

To evaluate the accuracy of SNP calling from resequencing of the Hanwoo genome, the same genomic DNA sample was applied to SNP chip analysis. We used BovineSNP50 BeadChip (Illumina) [57] to genotype the Hanwoo genome. In total, 40 proven bulls in the 45th Hanwoo Performance and Progeny Test Program in Korea, as well as 20 Angus and 19 Holstein individuals, were used for SNP genotyping with the same platform to investigate the ROHs. A consensus SNP genotype was obtained by selecting a maximally expressed genotype from the same location in a breed. Over 90% of the consensus genotypes appeared in more than half of the individuals for all three breeds (data not shown). A ROH was computed from the consensus SNP genotypes with the same criteria that were applied to calculate the ROH of whole-genome SNPs.

### Trait-associated genes and QTL regions

We obtained information on trait-associated genes from previous reports to analyze the Kuchinoshima-Ushi breed genome [7]. The genes were categorized into five economic traits: meat, disease resistance, growth, milk, and fecundity. Some genes that did not appear in NCBI RefSeq were added to the gene set for further analysis. QTL regions were identified from information on Cattle QTLs in the Animal QTLdb (Release 17; http://www.animalgenome.org/cgi-bin/QTLdb/BT/index) [20,21]. QTL locations by bp (UMD 3.1) were downloaded and three types of QTLs were selected: meat fat, meat tenderness, and milk traits. The associated names of these three QTL types described in QTLdb are as follows: intramuscular fat, marbling score, and marbling score (EBV) for meat fat; shear force and tenderness score for meat tenderness; and milk yield, milk yield (daughter deviation), milk yield (EBV), milk yield (PTA), dairy capacity composite index, and dairy form for milk traits.

Because the QTL were based on the UMD 3.1 genome, we converted the locations to coordinates from the Btau4.0 genome. Sequences of the selected QTL were extracted from UMD 3.1 genome sequences and aligned to the Btau4.0 reference genome sequences using LASTZ with the following options: seed = 14 of 22; chain = gapped, step = 5. The alignments were filtered with a minimum of 1,000 bases, 99% average identity, and 5% coverage. The syntenic locations were merged into a large location allowing gaps of 10% at the syntenic locations at most.

### Functional enrichment analysis

We determined genes whose genomic positions overlapped partially or completely with the ROH for each breed. We performed functional enrichment analysis against the candidate genes that were within a ROH region within the Gene Ontology and KEGG pathway terms using the Database for Annotation Visualization and Integrated Discovery (DAVID) tool (http://david.abcc.ncifcrf.gov/). Only the enriched GO terms with raw p-values <0.05 were used for further interpretation in this study. The functional relationships of the genes of interest were used in the Pathway studio program (Stratagene) [58].

## Abbreviations

Btau4.0: *Bos taurus* reference genome assembly build 4.0; CNV: Copy number variant; DAVID: Database for annotation visualization and integrated discovery; EBV: Estimated breeding value; EHH: Extended haplotype homozygosity; GATK: Genome analysis tool kit; Gb: Gigabase; gDNA: Genomic DNA; GO: Gene ontology; iHS: Integrated haplotype score; KEGG: Kyoto encyclopedia of genes and genomes; PTA: Predicted transmitting ability; QTL: Quantitative trait loci; RefSeq: Reference sequence database; ROH: Region of homozygosity; SNP: Single-nucleotide polymorphism; sROH: Specific ROH; UTR: Untranslated region.

## Competing interests

The authors declare that they have no competing interests.

## Authors’ contributions

KTL and THK designed and analyzed the data and wrote the manuscript. SYL and SA sequenced the Hanwoo genome and analyzed the data. SL and YHC collected and prepared Hanwoo genome samples. WHC, JK, SHL and NK analyzed the data and wrote the manuscript. GWJ, DL, and BK prepared the SNP chip data. JWC, XL, PS, and SM supplied the Black Angus and Holstein data, analyzed the data including Hanwoo genome, and revised the manuscript. SL prepared the genome browser and SNP submission. All authors read and approved the final manuscript.

## Supplementary Material

Additional file 1 **Read depth plot.** Distribution of the sequencing read depth for **(A)** Hanwoo, **(B)** Black Angus, and **(C)** Holstein. The horizontal axis shows the read depth mapped onto the same position of the reference genome. The read depth is considered to be the genome coverage (−fold). The vertical axis indicates the number of reads that belong to the depth. Click here for file

Additional file 2 **Sequencing read coverage.** Sequencing read coverage by chromosome for **(A)** Hanwoo, **(B)** Black Angus, and **(C)** Holstein. The horizontal axis indicates 30 chromosomes (excluding the Y chromosome and mitochondria) of the reference genome. Blue bars indicate the length of the reference chromosome and red bars indicate the region covered by the sequenced reads. The left vertical axis shows the Mbp scale of chromosome size. The green line indicates the percentage of sequencing read coverage. The right vertical axis shows the percentage scale of this coverage. Click here for file

Additional file 3 **Statistics of genetic variations.** General statistics for the sequencing reads and the genetic variations are shown. The variations were categorized separately by SNP, novel SNP, INDEL, and novel INDEL. In this case, “novel” means a variant that was not found in dbSNP 133. Click here for file

Additional file 4 **Concordance of SNPs.** The SNPs genotyped by the sequenced reads and the SNPs genotyped by SNP chip data were compared in the case of Hanwoo. Chip genotype indicates a genotype of the SNP chip and NGS genotype indicates a genotype of NGS data. “A” is reference allele, and “B” is an alternate allele. Click here for file

Additional file 5 **NS/SS/Is and ROHs (NGS/SNP chip) in 20,955 genes.** Gene locations, descriptions of genes and GO, trait-associated genes, ROHs from NGS data and SNP chip data in three breeds, and the number of NS/SS/Is including novel and damaging NS/SS/Is in 20,955 genes used in this study. Click here for file

Additional file 6 **Functional annotations of genetic variations in the reference nonredundant genes.** Genomic position, gene description, GO annotation, trait association, existence in ROHs from NGS data and SNP chip data in three breeds, and the number of NS/SS/Is including novel and damaging NS/SS/Is are described for each gene. Click here for file

Additional file 7 **ROH detection results from chip- and NGS-derived data of Hanwoo.** ROHs detected from chip and NGS data in the same Hanwoo individual. The upper portion is the result from chip data and the lower portion is from NGS data. Significant ROHs were detected by both platforms, and narrower ROHs were observed only in NGS-derived results. ROHs were identified from chip data using HomozygosityMapper [56] and from NGS data as described in the Methods. Click here for file

Additional file 8 Summary of genes residing in the ROHs of the three breeds. Click here for file

Additional file 9 **Summary of ROHs for the three breeds.** For each ROH region, the genomic position, chip ROH concordance, gene count, trait association, NS/SS/I variations, and breed-specific NS/SS/I variations are described. Click here for file

Additional file 10 **Results of functional enrichment analysis results using Gene Ontology (GO).** Excel spreadsheets of functional enrichment analyses on the basis of ‘Biological Processes’ GO annotation using DAVID for genes in the ROHs of the three breeds and for common genes in the ROHs of Hanwoo and Black Angus. Click here for file

## References

[B1] Tellam RL Lemay DG Van Tassell CP Lewin HA Worley KC Elsik CG Unlocking the bovine genome BMC Genomics 2009 10 193 10.1186/1471-2164-10-193 19393070 PMC2680899

[B2] Elsik CG Tellam RL Worley KC Gibbs RA Muzny DM Weinstock GM Adelson DL Eichler EE Elnitski L Guigo R The genome sequence of taurine cattle: a window to ruminant biology and evolution Science 2009 324 5926 522 528 19390049 10.1126/science.1169588 PMC2943200

[B3] Liu Y Qin X Song XZ Jiang H Shen Y Durbin KJ Lien S Kent MP Sodeland M Ren Y Bos taurus genome assembly BMC Genomics 2009 10 180 10.1186/1471-2164-10-180 19393050 PMC2686734

[B4] Zimin AV Delcher AL Florea L Kelley DR Schatz MC Puiu D Hanrahan F Pertea G Van Tassell CP Sonstegard TS A whole-genome assembly of the domestic cow, Bos taurus Genome Biol 2009 10 4 R42 10.1186/gb-2009-10-4-r42 19393038 PMC2688933

[B5] Van Tassell CP Smith TP Matukumalli LK Taylor JF Schnabel RD Lawley CT Haudenschild CD Moore SS Warren WC Sonstegard TS SNP discovery and allele frequency estimation by deep sequencing of reduced representation libraries Nat Methods 2008 5 3 247 252 10.1038/nmeth.1185 18297082

[B6] Eck SH Benet-Pages A Flisikowski K Meitinger T Fries R Strom TM Whole genome sequencing of a single Bos taurus animal for single nucleotide polymorphism discovery Genome Biol 2009 10 8 R82 10.1186/gb-2009-10-8-r82 19660108 PMC2745763

[B7] Kawahara-Miki R Tsuda K Shiwa Y Arai-Kichise Y Matsumoto T Kanesaki Y Oda S Ebihara S Yajima S Yoshikawa H Whole-genome resequencing shows numerous genes with nonsynonymous SNPs in the Japanese native cattle Kuchinoshima-Ushi BMC Genomics 2011 12 103 10.1186/1471-2164-12-103 21310019 PMC3048544

[B8] Stothard P Choi JW Basu U Sumner-Thomson JM Meng Y Liao X Moore SS Whole genome resequencing of black Angus and Holstein cattle for SNP and CNV discovery BMC Genomics 2011 12 559 10.1186/1471-2164-12-559 22085807 PMC3229636

[B9] Gibson J Morton NE Collins A Extended tracts of homozygosity in outbred human populations Hum Mol Genet 2006 15 5 789 795 10.1093/hmg/ddi493 16436455

[B10] Voight BF Kudaravalli S Wen X Pritchard JK A map of recent positive selection in the human genome PLoS Biol 2006 4 3 e72 10.1371/journal.pbio.0040072 16494531 PMC1382018

[B11] Weir BS Cardon LR Anderson AD Nielsen DM Hill WG Measures of human population structure show heterogeneity among genomic regions Genome Res 2005 15 11 1468 1476 10.1101/gr.4398405 16251456 PMC1310634

[B12] Sabeti PC Reich DE Higgins JM Levine HZ Richter DJ Schaffner SF Gabriel SB Platko JV Patterson NJ McDonald GJ Detecting recent positive selection in the human genome from haplotype structure Nature 2002 419 6909 832 837 10.1038/nature01140 12397357

[B13] Lee C Pollak EJ Genetic antagonism between body weight and milk production in beef cattle J Anim Sci 2002 80 2 316 321 11881921 10.2527/2002.802316x

[B14] Han SW The breed of cattle Breeds of Livestock 1996 1 Seoul: Sun-Jin publishing 148 160

[B15] McKenna A Hanna M Banks E Sivachenko A Cibulskis K Kernytsky A Garimella K Altshuler D Gabriel S Daly M The genome analysis toolkit: a MapReduce framework for analyzing next-generation DNA sequencing data Genome Res 2010 20 9 1297 1303 10.1101/gr.107524.110 20644199 PMC2928508

[B16] DePristo MA Banks E Poplin R Garimella KV Maguire JR Hartl C Philippakis AA del Angel G Rivas MA Hanna M A framework for variation discovery and genotyping using next-generation DNA sequencing data Nat Genet 2011 43 5 491 498 10.1038/ng.806 21478889 PMC3083463

[B17] Decker JE Pires JC Conant GC McKay SD Heaton MP Chen K Cooper A Vilkki J Seabury CM Caetano AR Resolving the evolution of extant and extinct ruminants with high-throughput phylogenomics Proc Natl Acad Sci USA 2009 106 44 18644 18649 10.1073/pnas.0904691106 19846765 PMC2765454

[B18] Marquez B Ameye G Vallet CM Tulkens PM Poirel HA Van Bambeke F Characterization of Abcc4 gene amplification in stepwise-selected mouse J774 macrophages resistant to the topoisomerase II inhibitor ciprofloxacin PLoS One 2011 6 12 e28368 10.1371/journal.pone.0028368 22162766 PMC3230599

[B19] Schaeren-Wiemers N Bonnet A Erb M Erne B Bartsch U Kern F Mantei N Sherman D Suter U The raft-associated protein MAL is required for maintenance of proper axon–glia interactions in the central nervous system J Cell Biol 2004 166 5 731 742 10.1083/jcb.200406092 15337780 PMC2172435

[B20] Hu ZL Fritz ER Reecy JM AnimalQTLdb: a livestock QTL database tool set for positional QTL information mining and beyond Nucleic Acids Res 2007 35 Database issue D604 D609 17135205 10.1093/nar/gkl946 PMC1781224

[B21] Hu ZL Reecy JM Animal QTLdb: beyond a repository. A public platform for QTL comparisons and integration with diverse types of structural genomic information Mamm Genome 2007 18 1 1 4 10.1007/s00335-006-0105-8 17245610

[B22] Sasaki Y Nagai K Nagata Y Doronbekov K Nishimura S Yoshioka S Fujita T Shiga K Miyake T Taniguchi Y Exploration of genes showing intramuscular fat deposition-associated expression changes in musculus longissimus muscle Anim Genet 2006 37 1 40 46 10.1111/j.1365-2052.2005.01380.x 16441294

[B23] Yamada T Sasaki S Sukegawa S Yoshioka S Takahagi Y Morita M Murakami H Morimatsu F Fujita T Miyake T Association of a single nucleotide polymorphism in titin gene with marbling in Japanese Black beef cattle BMC Res Notes 2009 2 78 10.1186/1756-0500-2-78 19419586 PMC2683863

[B24] Labeit S Kolmerer B Titins: giant proteins in charge of muscle ultrastructure and elasticity Science 1995 270 5234 293 296 10.1126/science.270.5234.293 7569978

[B25] Ku CS Naidoo N Teo SM Pawitan Y Regions of homozygosity and their impact on complex diseases and traits Hum Genet 2011 129 1 1 15 10.1007/s00439-010-0920-6 21104274

[B26] Weidemann JF Kaess G Carrljthers LD The histology of pre-rigor and post-rigor ox muscle before and after cooking and its relation to tenderness J Food Sci 1967 32 1 7 13 10.1111/j.1365-2621.1967.tb01946.x

[B27] Solomon C White JH Kremer R Mitogen-activated protein kinase inhibits 1,25-dihydroxyvitamin D3-dependent signal transduction by phosphorylating human retinoid X receptor alpha J Clin Invest 1999 103 12 1729 1735 10.1172/JCI6871 10377179 PMC408392

[B28] Lim D Kim NK Park HS Lee SH Cho YM Oh SJ Kim TH Kim H Identification of candidate genes related to bovine marbling using protein-protein interaction networks Int J Biol Sci 2011 7 7 992 1002 21912507 10.7150/ijbs.7.992 PMC3164149

[B29] Huq MD Tsai NP Lin YP Higgins L Wei LN Vitamin B6 conjugation to nuclear corepressor RIP140 and its role in gene regulation Nat Chem Biol 2007 3 3 161 165 10.1038/nchembio861 17277785

[B30] Brandes R Arad R Bar-Tana J Inducers of adipose conversion activate transcription promoted by a peroxisome proliferators response element in 3T3-L1 cells Biochem Pharmacol 1995 50 11 1949 1951 10.1016/0006-2952(95)02082-9 8615877

[B31] Kuhn C Weikard R An investigation into the genetic background of coat colour dilution in a Charolais x German Holstein F2 resource population Anim Genet 2007 38 2 109 113 10.1111/j.1365-2052.2007.01569.x 17302792

[B32] Abu Safieh L Aldahmesh MA Shamseldin H Hashem M Shaheen R Alkuraya H Al Hazzaa SA Al-Rajhi A Alkuraya FS Clinical and molecular characterisation of Bardet-Biedl syndrome in consanguineous populations: the power of homozygosity mapping J Med Genet 2010 47 4 236 241 10.1136/jmg.2009.070755 19858128

[B33] Collin RW Safieh C Littink KW Shalev SA Garzozi HJ Rizel L Abbasi AH Cremers FP den Hollander AI Klevering BJ Mutations in C2ORF71 cause autosomal-recessive retinitis pigmentosa Am J Hum Genet 2010 86 5 783 788 10.1016/j.ajhg.2010.03.016 20398884 PMC2869006

[B34] Harville HM Held S Diaz-Font A Davis EE Diplas BH Lewis RA Borochowitz ZU Zhou W Chaki M MacDonald J Identification of 11 novel mutations in eight BBS genes by high-resolution homozygosity mapping J Med Genet 2010 47 4 262 267 10.1136/jmg.2009.071365 19797195 PMC3017466

[B35] Iseri SU Wyatt AW Nurnberg G Kluck C Nurnberg P Holder GE Blair E Salt A Ragge NK Use of genome-wide SNP homozygosity mapping in small pedigrees to identify new mutations in VSX2 causing recessive microphthalmia and a semidominant inner retinal dystrophy Hum Genet 2010 128 1 51 60 10.1007/s00439-010-0823-6 20414678

[B36] Lapunzina P Aglan M Temtamy S Caparros-Martin JA Valencia M Leton R Martinez-Glez V Elhossini R Amr K Vilaboa N Identification of a frameshift mutation in Osterix in a patient with recessive osteogenesis imperfecta Am J Hum Genet 2010 87 1 110 114 10.1016/j.ajhg.2010.05.016 20579626 PMC2896769

[B37] Nicolas E Poitelon Y Chouery E Salem N Levy N Megarbane A Delague V CAMOS, a nonprogressive, autosomal recessive, congenital cerebellar ataxia, is caused by a mutant zinc-finger protein, ZNF592 Eur J Hum Genet 2010 18 10 1107 1113 10.1038/ejhg.2010.82 20531441 PMC2987462

[B38] Pang J Zhang S Yang P Hawkins-Lee B Zhong J Zhang Y Ochoa B Agundez JA Voelckel MA Fisher RB Loss-of-function mutations in HPSE2 cause the autosomal recessive urofacial syndrome Am J Hum Genet 2010 86 6 957 962 10.1016/j.ajhg.2010.04.016 20560209 PMC3032074

[B39] Uz E Alanay Y Aktas D Vargel I Gucer S Tuncbilek G von Eggeling F Yilmaz E Deren O Posorski N Disruption of ALX1 causes extreme microphthalmia and severe facial clefting: expanding the spectrum of autosomal-recessive ALX-related frontonasal dysplasia Am J Hum Genet 2010 86 5 789 796 10.1016/j.ajhg.2010.04.002 20451171 PMC2869009

[B40] Walsh T Shahin H Elkan-Miller T Lee MK Thornton AM Roeb W Abu Rayyan A Loulus S Avraham KB King MC Whole exome sequencing and homozygosity mapping identify mutation in the cell polarity protein GPSM2 as the cause of nonsyndromic hearing loss DFNB82 Am J Hum Genet 2010 87 1 90 94 10.1016/j.ajhg.2010.05.010 20602914 PMC2896776

[B41] Lencz T Lambert C DeRosse P Burdick KE Morgan TV Kane JM Kucherlapati R Malhotra AK Runs of homozygosity reveal highly penetrant recessive loci in schizophrenia Proc Natl Acad Sci USA 2007 104 50 19942 19947 10.1073/pnas.0710021104 18077426 PMC2148402

[B42] Nalls MA Guerreiro RJ Simon-Sanchez J Bras JT Traynor BJ Gibbs JR Launer L Hardy J Singleton AB Extended tracts of homozygosity identify novel candidate genes associated with late-onset Alzheimer’s disease Neurogenetics 2009 10 3 183 190 10.1007/s10048-009-0182-4 19271249 PMC2908484

[B43] Yang TL Guo Y Zhang LS Tian Q Yan H Papasian CJ Recker RR Deng HW Runs of homozygosity identify a recessive locus 12q21.31 for human adult height J Clin Endocrinol Metab 2010 95 8 3777 3782 10.1210/jc.2009-1715 20466785 PMC2913044

[B44] Barendse W Bunch RJ Thomas MB Harrison BE A splice site single nucleotide polymorphism of the fatty acid binding protein 4 gene appears to be associated with intramuscular fat deposition in longissimus muscle in Australian cattle Anim Genet 2009 40 5 770 773 10.1111/j.1365-2052.2009.01913.x 19466936

[B45] Hoashi S Hinenoya T Tanaka A Ohsaki H Sasazaki S Taniguchi M Oyama K Mukai F Mannen H Association between fatty acid compositions and genotypes of FABP4 and LXR-alpha in Japanese black cattle BMC Genet 2008 9 84 19077218 10.1186/1471-2156-9-84 PMC2628680

[B46] Cho S Park TS Yoon DH Cheong HS Namgoong S Park BL Lee HW Han CS Kim EM Cheong IC Identification of genetic polymorphisms in FABP3 and FABP4 and putative association with back fat thickness in Korean native cattle BMB Rep 2008 41 1 29 34 10.5483/BMBRep.2008.41.1.029 18304447

[B47] Lee SH van der Werf JH Park EW Oh SJ Gibson JP Thompson JM Genetic polymorphisms of the bovine fatty acid binding protein 4 gene are significantly associated with marbling and carcass weight in Hanwoo (Korean Cattle) Anim Genet 2010 41 4 442 444 20331595 10.1111/j.1365-2052.2010.02024.x

[B48] Laliotis GP Bizelis I Rogdakis E Comparative approach of the de novo fatty acid synthesis (Lipogenesis) between ruminant and non ruminant mammalian species: from biochemical level to the main regulatory lipogenic genes Curr Genomics 2010 11 3 168 183 10.2174/138920210791110960 21037855 PMC2878982

[B49] Lee SH Kim SC Choi BH Lim D Kim NK Lee JH Kim OH Lee CS Kim HC Yang BS mt-COX1, mt-ND1 and CREBP are indicators of intramuscular fat content in Hanwoo (Korean cattle) Livest Sci 2012 146 160 167 10.1016/j.livsci.2012.03.003

[B50] Yamamoto KK Gonzalez GA Biggs WH 3rd Montminy MR Phosphorylation-induced binding and transcriptional efficacy of nuclear factor CREB Nature 1988 334 6182 494 498 10.1038/334494a0 2900470

[B51] Casimir DA Ntambi JM cAMP activates the expression of stearoyl-CoA desaturase gene 1 during early preadipocyte differentiation J Biol Chem 1996 271 47 29847 29853 10.1074/jbc.271.47.29847 8939924

[B52] Wang YH Bower NI Reverter A Tan SH De Jager N Wang R McWilliam SM Cafe LM Greenwood PL Lehnert SA Gene expression patterns during intramuscular fat development in cattle J Anim Sci 2009 87 1 119 130 18820161 10.2527/jas.2008-1082

[B53] Dooley KA Bennett MK Osborne TF A critical role for CREB as a co-activator in sterol regulated transcription of HMG CoA synthase promoter J Biol Chem 1999 274 5285 5291 10.1074/jbc.274.9.5285 10026135

[B54] Homer N Merriman B Nelson SF BFAST: an alignment tool for large scale genome resequencing PLoS One 2009 4 11 e7767 10.1371/journal.pone.0007767 19907642 PMC2770639

[B55] Kumar P Henikoff S Ng PC Predicting the effects of coding non-synonymous variants on protein function using the SIFT algorithm Nat Protoc 2009 4 7 1073 1081 19561590 10.1038/nprot.2009.86

[B56] Seelow D Schuelke M Hildebrandt F Nurnberg P HomozygosityMapper--an interactive approach to homozygosity mapping Nucleic Acids Res 2009 37 Web Server issue W593 W599 19465395 10.1093/nar/gkp369 PMC2703915

[B57] Matukumalli LK Lawley CT Schnabel RD Taylor JF Allan MF Heaton MP O’Connell J Moore SS Smith TP Sonstegard TS Development and characterization of a high density SNP genotyping assay for cattle PLoS One 2009 4 4 e5350 10.1371/journal.pone.0005350 19390634 PMC2669730

[B58] Nikitin A Egorov S Daraselia N Mazo I Pathway studio–the analysis and navigation of molecular networks Bioinformatics 2003 19 16 2155 10.1093/bioinformatics/btg290 14594725

