# Supplementary figures and images for: Whole-genome resequencing of Hanwoo (Korean cattle) and insight into regions of homozygosity
Source: BMC Genomics. 2013 Jul 30;14:519. doi: 10.1186/1471-2164-14-519 (PMC3750754; doi:10.1186/1471-2164-14-519)

## Slide 1
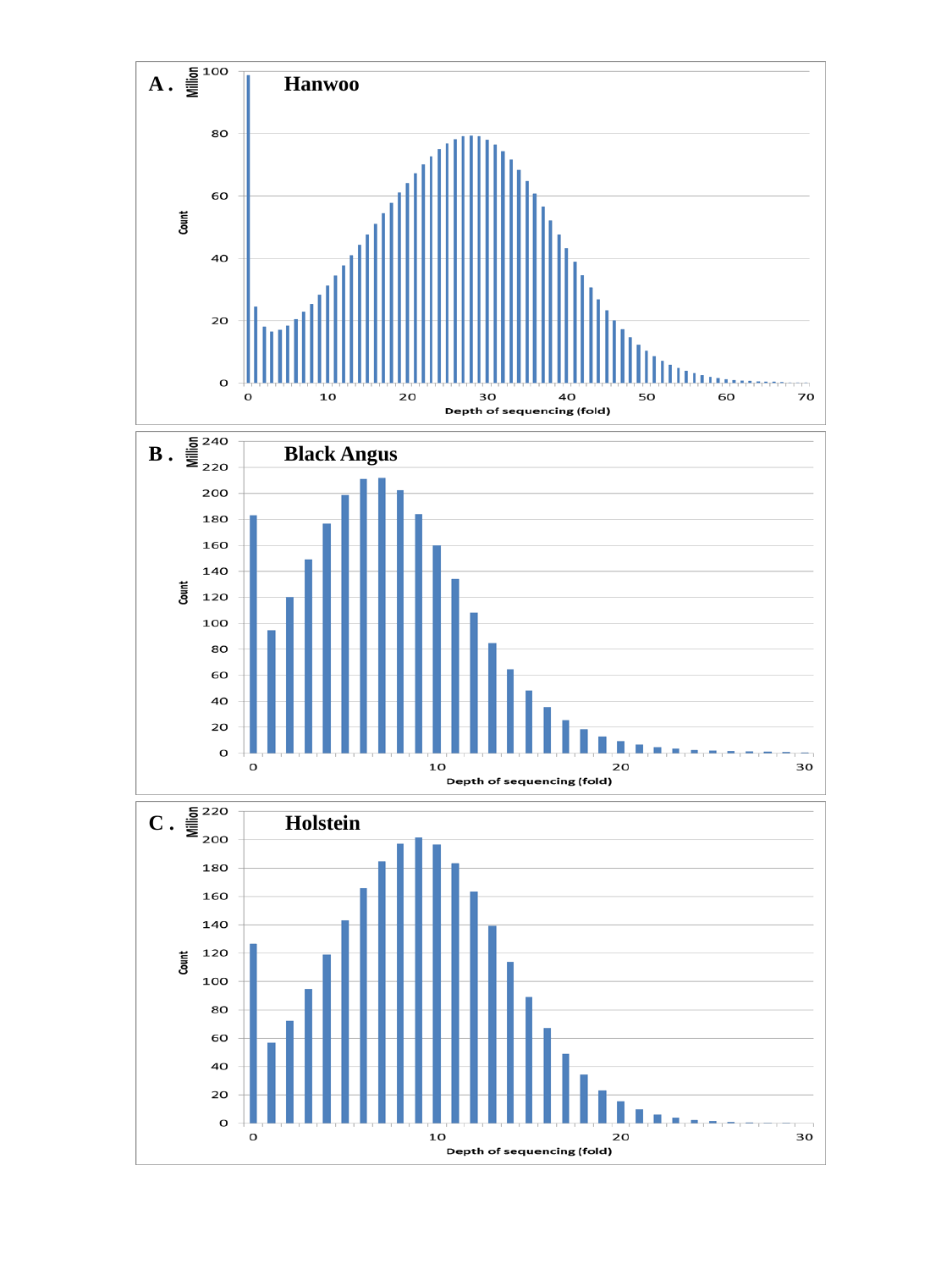

A . Hanwoo
B . Black Angus
C . Holstein

Supplement: Additional file 1 — Read depth plot. Distribution of the sequencing read depth for (A) Hanwoo, (B) Black Angus, and (C) Holstein. The horizontal axis shows the read depth mapped onto the same position of the reference genome. The read depth is considered to be the genome coverage (−fold). The vertical axis indicates the number of reads that belong to the depth. [file 1471-2164-14-519-S1.pptx]

## Slide 1
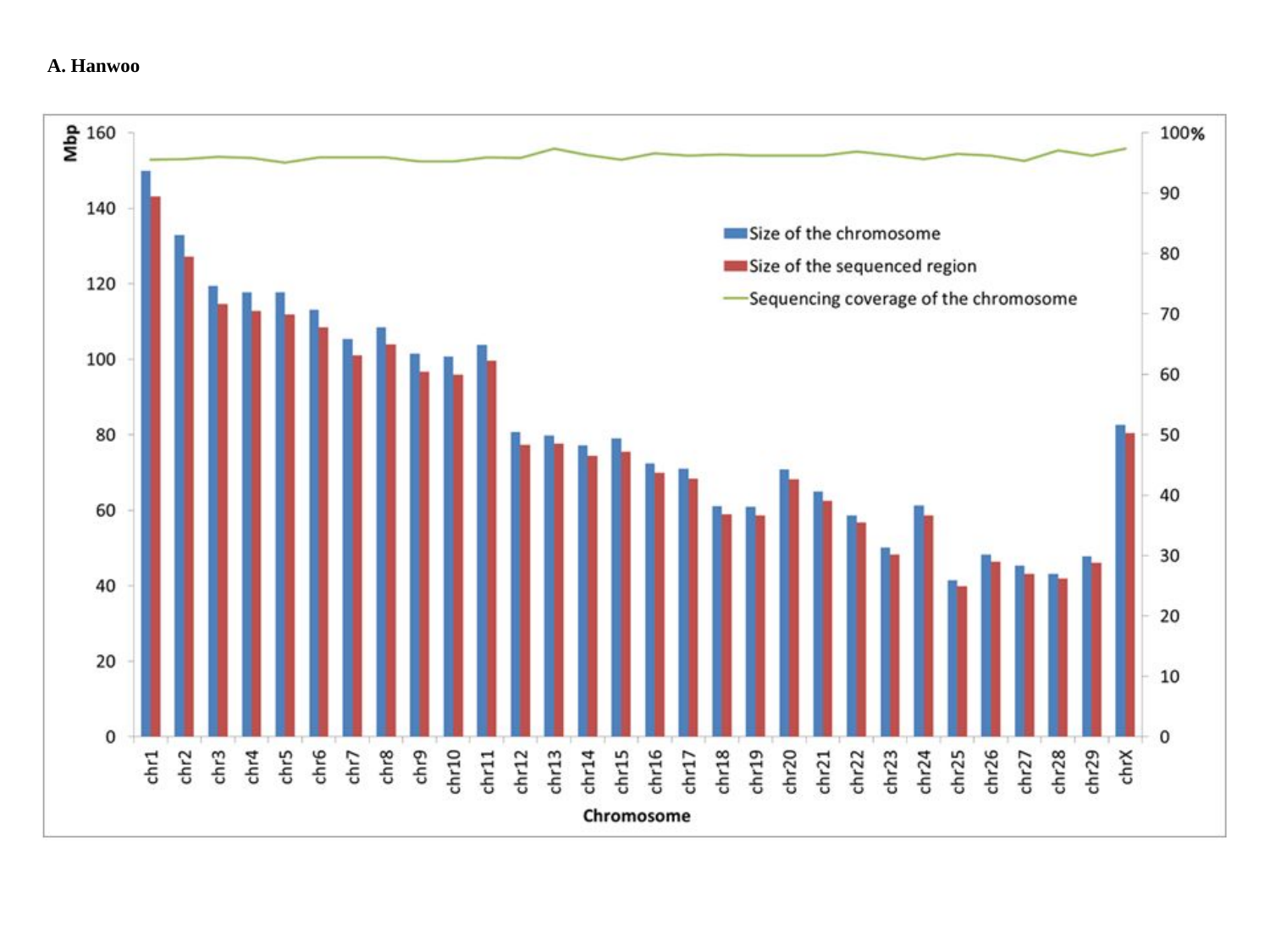

A. Hanwoo

## Slide 2
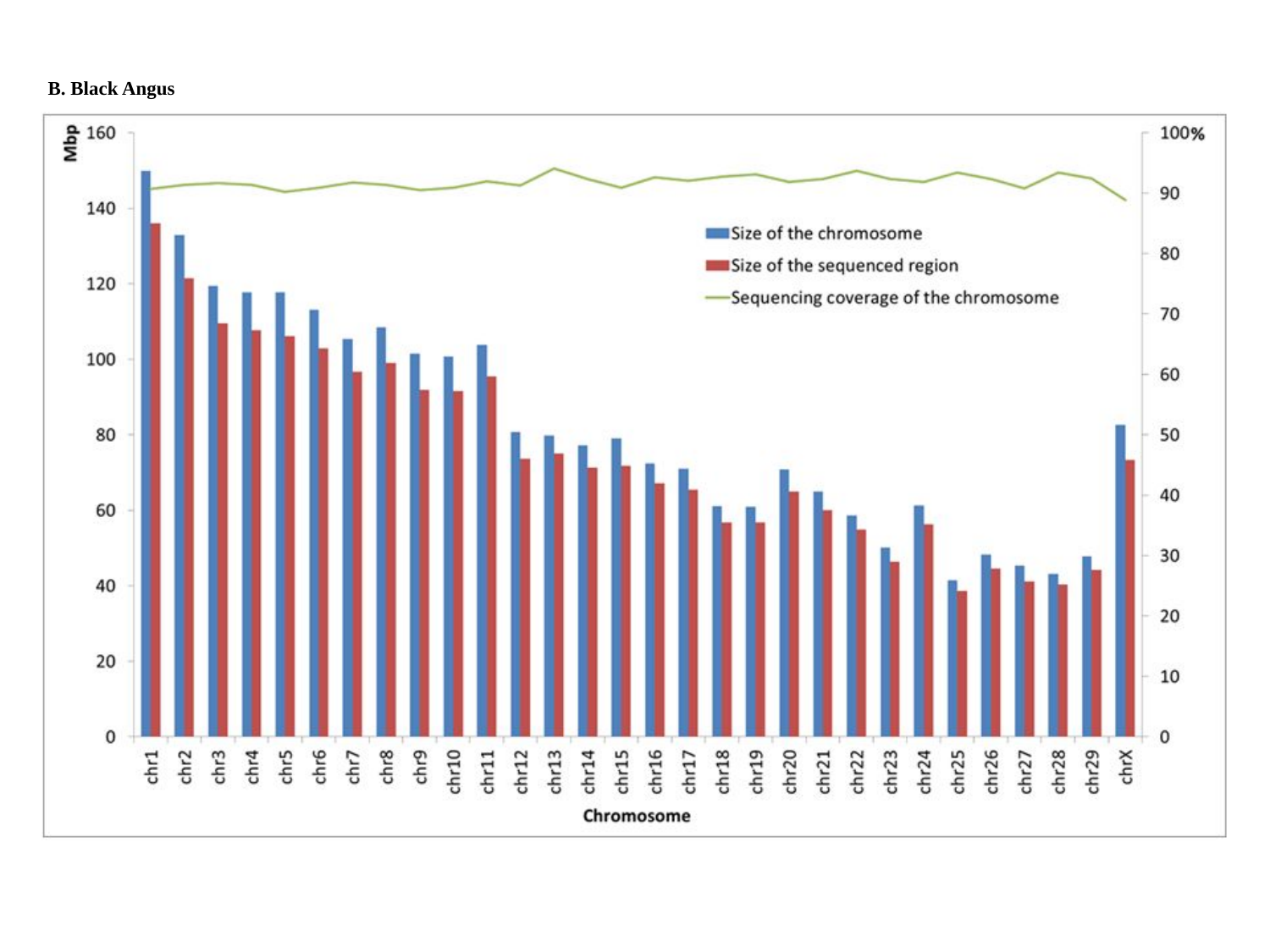

B. Black Angus

## Slide 3
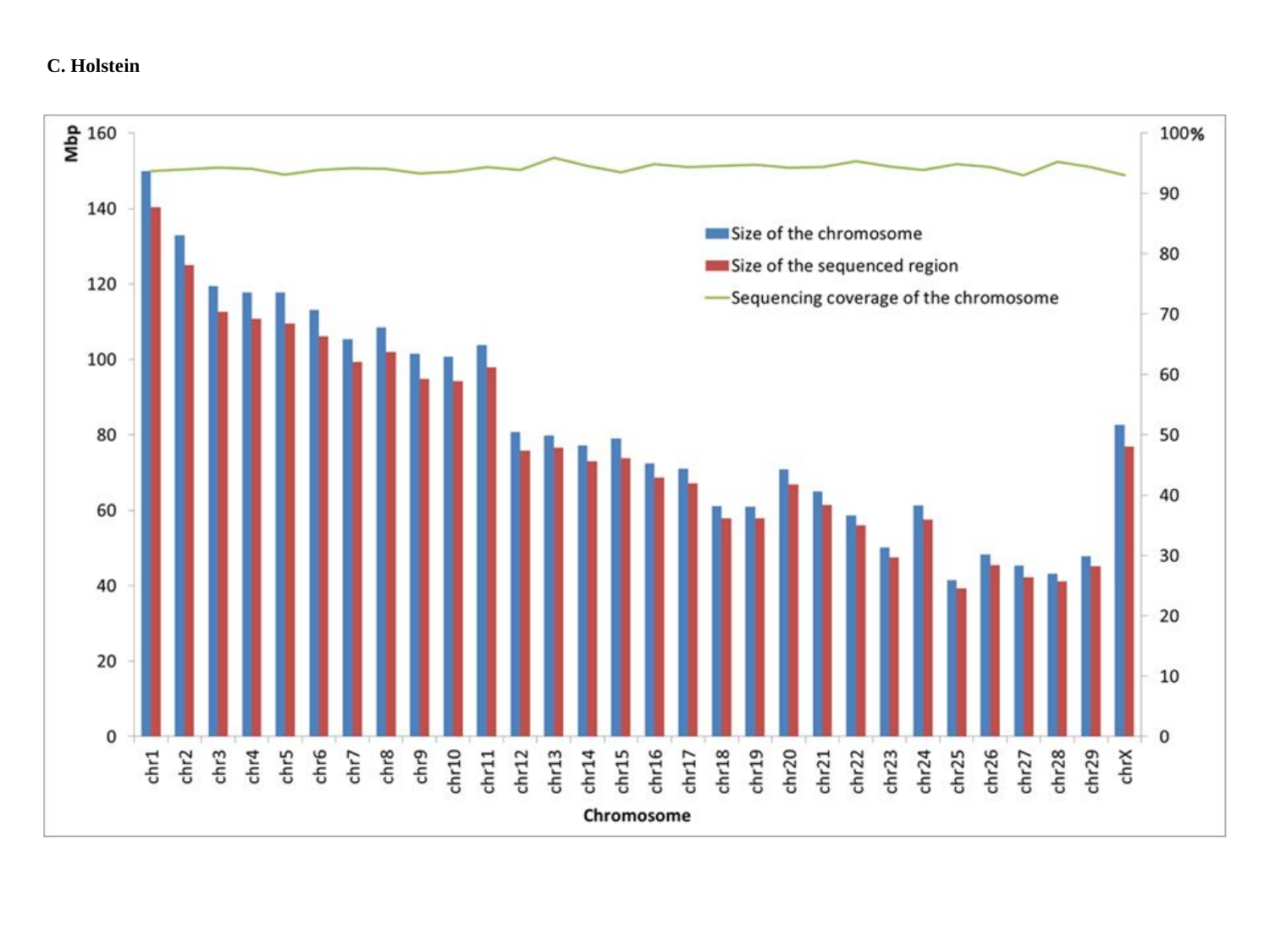

C. Holstein

Supplement: Additional file 2 — Sequencing read coverage. Sequencing read coverage by chromosome for (A) Hanwoo, (B) Black Angus, and (C) Holstein. The horizontal axis indicates 30 chromosomes (excluding the Y chromosome and mitochondria) of the reference genome. Blue bars indicate the length of the reference chromosome and red bars indicate the region covered by the sequenced reads. The left vertical axis shows the Mbp scale of chromosome size. The green line indicates the percentage of sequencing read coverage. The right vertical axis shows the percentage scale of this coverage. [file 1471-2164-14-519-S2.pptx]

## Slide 1
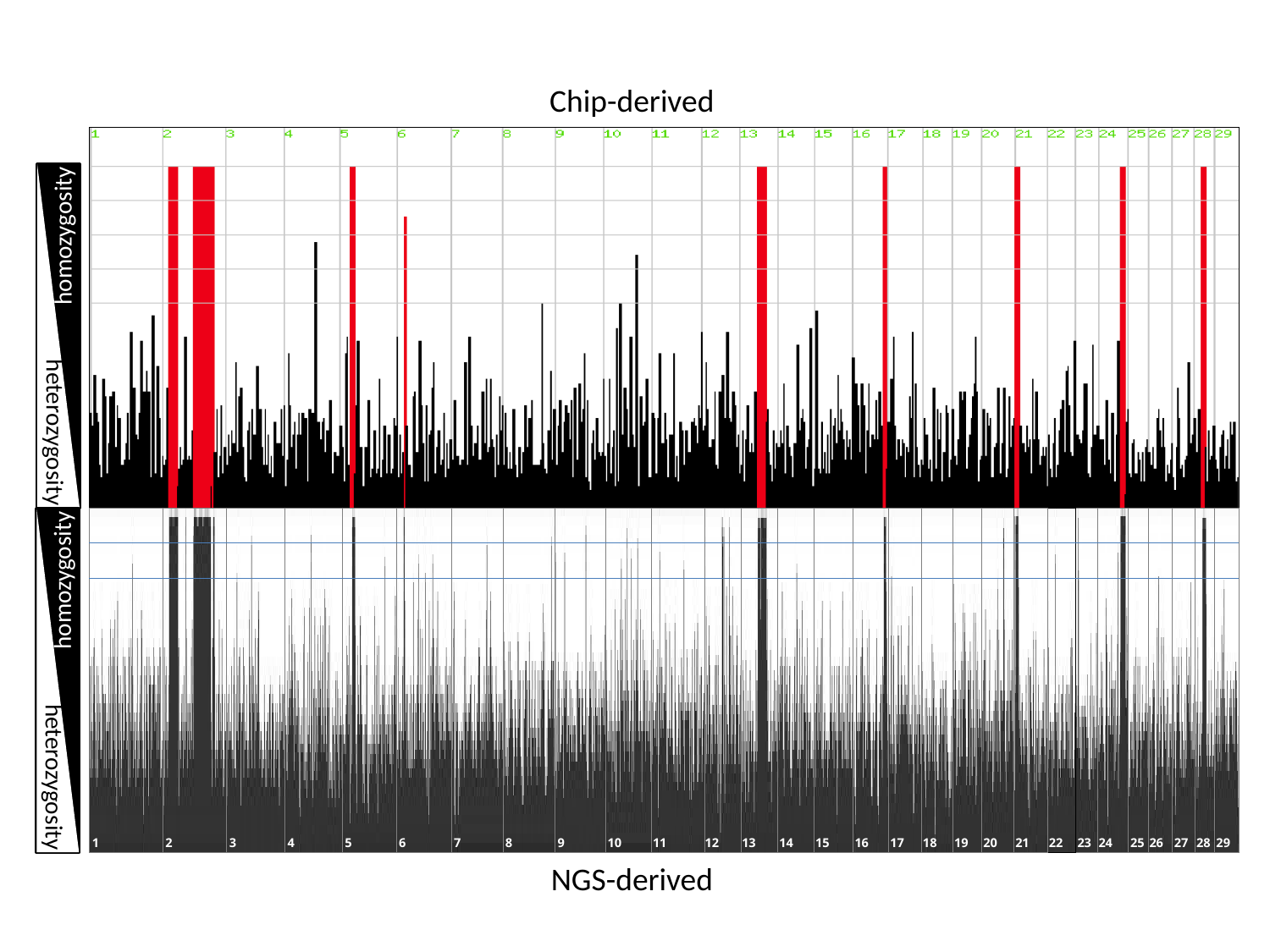

Chip-derived
homozygosity
heterozygosity
26
23
22
21
20
18
17
16
13
3
1
2
4
5
6
7
8
9
10
11
12
14
15
19
24
25
27
28
29
homozygosity
heterozygosity
NGS-derived

Supplement: Additional file 7 — ROH detection results from chip- and NGS-derived data of Hanwoo. ROHs detected from chip and NGS data in the same Hanwoo individual. The upper portion is the result from chip data and the lower portion is from NGS data. Significant ROHs were detected by both platforms, and narrower ROHs were observed only in NGS-derived results. ROHs were identified from chip data using HomozygosityMapper [56] and from NGS data as described in the Methods. [file 1471-2164-14-519-S7.pptx]

## Slide 1
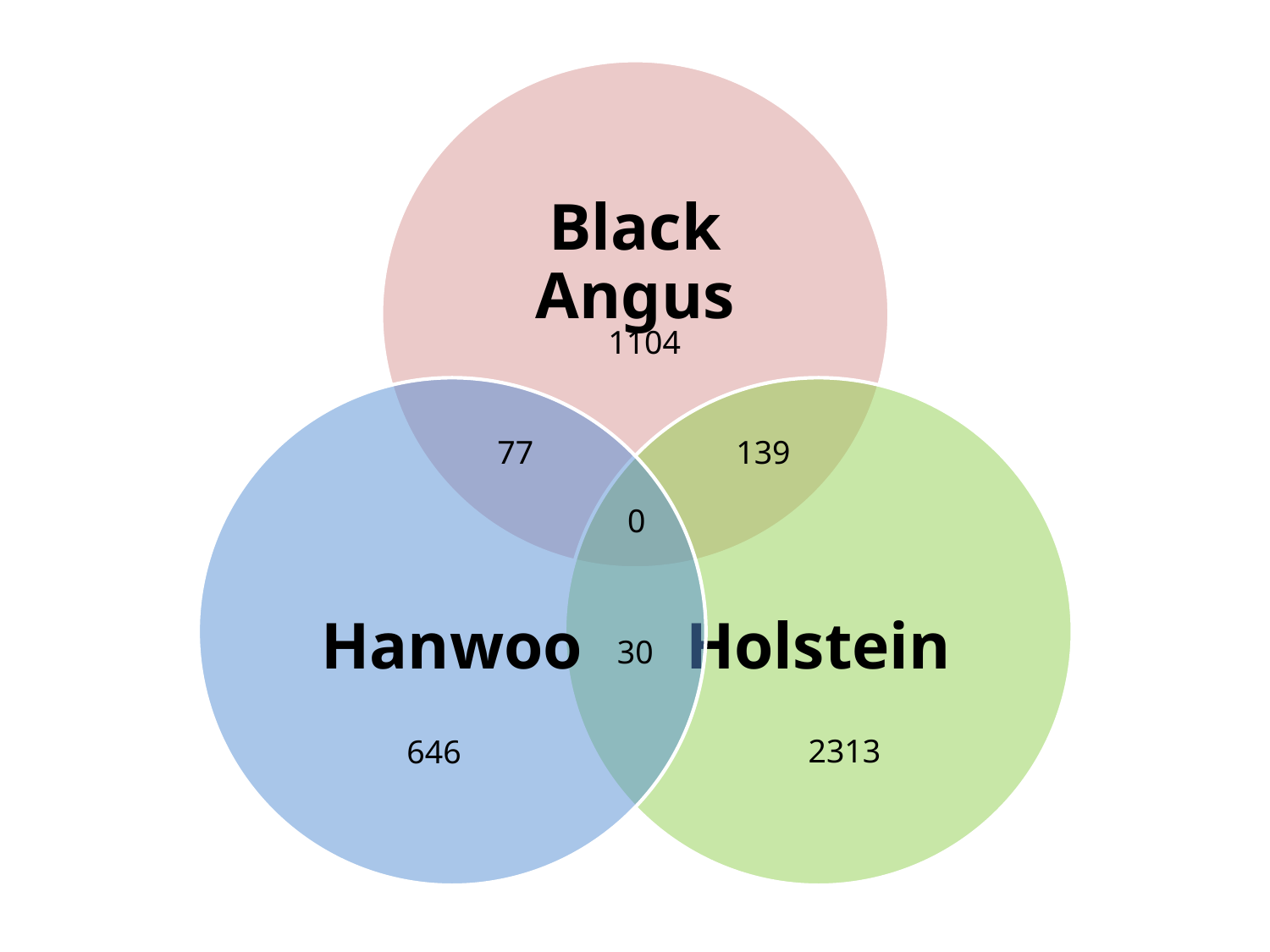

1104
77
139
0
30
2313
646

Supplement: Additional file 8 — Summary of genes residing in the ROHs of the three breeds. [file 1471-2164-14-519-S8.pptx]
