# Supplementary material for: Whole-genome resequencing of Hanwoo (Korean cattle) and insight into regions of homozygosity
Source: BMC Genomics. 2013 Jul 30;14:519. doi: 10.1186/1471-2164-14-519 (PMC3750754; doi:10.1186/1471-2164-14-519)
Supplement: Additional file 4 — Concordance of SNPs. The SNPs genotyped by the sequenced reads and the SNPs genotyped by SNP chip data were compared in the case of Hanwoo. Chip genotype indicates a genotype of the SNP chip and NGS genotype indicates a genotype of NGS data. “A” is reference allele, and “B” is an alternate allele. [file 1471-2164-14-519-S4.docx]

Additional File 4. Concordance of SNPs

|  |  | **Chip genotype** | | | | | |
| --- | --- | --- | --- | --- | --- | --- | --- |
|  |  | A/A | | A/B | | B/B | |
| **NGS**  **genotype** | A/A | 24,165 | (99.1%) | 464 | (3.8%) | 226 | (1.7%) |
|  | A/B | 186 | (0.8%) | 11,818 | (95.6%) | 875 | (6.4%) |
|  | B/B | 9 | (0.0%) | 62 | (0.5%) | 12,499 | (91.5%) |
| **total** | | **24,391** | | **12,362** | | **13,658** | |

Overall genotype concordance (96.2%), Non-reference sensitivity (97.1%), Non-reference discrepancy rate (7.0%)
